# Low catestatin as a risk factor for cardiovascular disease – assessment in patients with adrenal incidentalomas

**DOI:** 10.3389/fendo.2023.1198911

**Published:** 2023-07-14

**Authors:** Ewa Zalewska, Piotr Kmieć, Jakub Sobolewski, Andrzej Koprowski, Krzysztof Sworczak

**Affiliations:** ^1^ Department of Endocrinology and Internal Medicine, Medical University of Gdańsk, Gdańsk, Poland; ^2^ First Department of Cardiology, Medical University of Gdańsk, Gdańsk, Poland

**Keywords:** catestatin, adrenal incidentaloma (AI), cardiovascular disease(s), risk predictor, metabolic syndrome

## Abstract

**Background:**

Catestatin (Cts) is a peptide derived from proteolytic cleavage of chromogranin A, which exhibits cardioprotective and anti-inflammatory properties. Cts has been proposed as a potential biomarker for cardiovascular (CV) disease.

**Objectives:**

examining Cts in patients with incidentally discovered adrenocortical adenomas (AI), and its associations with CV risk factors and blood pressure (BP).

**Materials and methods:**

In this cross-sectional study, 64 AI patients without overt CV disease other than primary hypertension were recruited along with 24 age-, sex-, and body-mass-index (BMI)-matched controls with normal adrenal morphology. Laboratory, 24-h ambulatory BP monitoring, echocardiography, and common carotid artery sonography examinations were performed.

**Results:**

Unadjusted Cts was higher in AI patients (median 6.5, interquartile range: 4.9-37 ng/ml) versus controls (4.5 (3.5 – 28)), p=0.048, however, the difference was insignificant after adjusting for confounding variables. Cts was lower in subjects with metabolic syndrome than in those without it (5.2 (3.9- 6.9) vs. 25.7 (5.8-115) ng/ml, p<0.01), and in men compared to women (4.9 (4-7.4) ng/ml vs. 7 (4.8-100), p=0.015). AI patients in the lower half of Cts levels compared to those in the upper had a higher prevalence of hypertension (OR 0.15, 95% CI: 0.041-0.5, p<0.001) and metabolic syndrome (OR 0.15, 95% CI 0.041-0.5, p<0.001). In AI patients Cts correlated positively with high-density lipoprotein cholesterol (Spearman’s r=0.31), negatively with BMI (r=-0.31), and 10-year atherosclerotic CV disease risk (r=-0.42).

**Conclusions:**

Our data indicate associations between CV risk factors and Cts. More clinical research is needed to apply serum Cts as a biomarker.

## 1 Introduction

Risk factors for atherosclerotic cardiovascular disease (ASCVD) can be divided into nonmodifiable (e.g. age or sex) and modifiable (smoking, elevated blood pressure (BP), dyslipidemia, diabetes (DM), and obesity). Apart from established risk factors, new are sought (e.g. uric acid (UA) (1) and high-sensitivity C-reactive protein [hs-CRP) (2)]) to help distinguish persons at higher risk of developing cardiovascular disease (CVD), who would benefit more from medical interventions such as low-density lipoprotein cholesterol (LDL-C) reduction (3).

The sympathetic nervous system plays a pivotal role in CVD development. Chromogranin A (CgA) is co-stored and co-released with catecholaminec from sympathetic neuronal vesicles and the adrenall medulla. One of CgA's proteolytic cleavage products is catestatin (Cts), a cardioprotective, anti-hypertensive, and anti-inflammatory peptide (4, 5). *In vitro*, Cts was shown to bind to nicotinic acetylcholine receptors, which inhibits membrane depolarization and blocks calcium influx, and, consequently, suppresses catecholamine release and activation of the sympathetic nervous system (6). Studies with animal models demonstrated Cts exerts anti-inflammatory effects, cardioprotection, and reduces obesity and insulin resistance (7–9). Clinical studies indicate Cts is involved in the course of hypertension (HT), coronary artery diseases (CAD), and heart failure (HF) (10–12). Adolescents with metabolic syndrome (MetS) had decreased Cts, which was postulated as a novel CVD risk factor (12–14).

In the current study we aimed at 1) determining Cts levels in patients with an incidentally-discovered adrenocortical adenoma/hyperplasia (AI) and without overt CVD other than HT, as well as 2) investigating associations between Cts and ASCVD risk modifiers, and asymptomatic HT-mediated organ damage (15). The presence of an AI *per se*, and particularly mild autonomous cortisol secretion (MACS) in its course, have been associated with metabolic disorders, elevated CV risk and mortality (16). So far, Cts has not been investigated in this patient population.

## 2 Subjects and methods

### 2.1 Study population

Study participants with an AI were recruited among 376 consecutive adult patients hospitalized in the Department of Endocrinology and Internal Medicine of the University Clinical Center of the Medical University of Gdańsk between November 2018 through February 2020 due to an adrenal lesion. We included 64 patients with radiological features of an adrenal adenoma/hyperplasia revealed by computed tomography (CT) or magnetic resonance (MR), who agreed to participate in the study, and met none of the following exclusion criteria: 1) age over 75 or under 40; 2) obesity grade III (BMI >40 kg/m^2^); 3) premenopausal period; 4) adrenal hormone excess other than MACS, i.e. cortisolemia between 50 and 138 nmol/l in the overnight 1-mg dexamethasone suppression test (DST) and no phenotypic features of Cushing’s syndrome (17); 5) kidney disease with eGFR<60 ml/min/1.73m^2^ and/or proteinuria >0.25 g/24h); 6) treatment with a mineralocorticoid receptor antagonist; 7) established and/or overt CVD other than primary HT, including: a) ASCVD (CAD, stroke, transient ischemic attack, peripheral artery disease), b) significant cardiac disease (e.g. pathological arrhythmia, severe valvular heart disease, cardiac tamponade, cardiomyopathy, congenital heart disease, HF), c) vascular diseases (among others venous thromboembolism and vasculitis); 8) active malignancy; 9) decompensated autoimmune disease or immune disease associated with CV and/or renal complications; 10) infectious diseases; 11) current or past addiction to alcohol and/or illicit drugs. Study participants were recruited based on anamnesis, physical examination, additional examinations available for review prior to enrollment and performed in the course of the study. Initially, 73 patients were included, however, three withdrew their consent to participate due to the COVID-19 pandemic, in four patients transthoracic echocardiography (TTE) revealed cardiac post-ischemic lesions, and two were diagnosed with primary aldosteronism.

Based on medical records of our hospital, which included examinations ordered in outpatient clinics and the emergency department, we identified 153 persons with normal adrenal morphology in a CT/MRI scan performed within five years preceding this study. There were 129 who met at least one of the above-listed exclusion criteria, declined participation or were unreachable, therefore, 24 subjects without an AI were enrolled as controls.

The research complied with the Declaration of Helsinki and was approved by the Independent Bioethics Committee for Research of our University. Informed consent for inclusion in the study was obtained in writing from each participant.

### 2.2 Study design

Both AI patients and controls underwent the following evaluation: 1) medical interview; 2) physical examination; 3) antecubital venous blood sampling for laboratory analyses; 4) resting 12-lead electrocardiography (ECG); 5) TTE; 6) common carotid artery (CCA) ultrasonography (USG) including CIMT determination, 7) 24-hour ambulatory blood pressure monitoring (ABPM).

Body-mass-index (BMI) was calculated by dividing body weight (W) in kg by the square of height (H) in meters. 2009 International Diabetes Federation criteria were used to diagnose MetS (18). Subjects with HT received hypotensive medications at the time of enrollment or were diagnosed by ABPM based on mean systolic and diastolic BP (SBP and DBP, respectively) of at least 135/85 mmHg for daytime, 120/75 mmHg for nighttime, and/or 130/80 mmHg for the 24-h period (15). Atherogenic dyslipidemia was defined as triglycerides (TGL) ≥150 mg/dL and serum high-density lipoprotein cholesterol (HDL-C) <40 mg/dL for men and <45 mg/dL for women.

In all study participants, 10-year ACSVD risk was estimated using the 2018 calculator provided online by the American Heart Association and the American College of Cardiology based on Framingham Heart Study (FHS-ASCVD Risk) (19, 20). The calculator estimates 10-year risk of developing ASCVD including coronary death, myocardial infarction, coronary insufficiency, angina, ischemic stroke, hemorrhagic stroke, transient ischemic attack, peripheral artery disease, and HF for individuals aged 30 to 74 and without CVD at baseline based on the following predictors: age, type 2 DM (DMt2), smoking, treated and untreated SBP, total cholesterol (TC), HDL-C, and LDL-C.

For nondiabetic subjects, 10-year CVD risk was also calculated using Systematic Coronary Risk Estimation 2 (SCORE2) for subjects aged 40-69 and SCORE2-Older People (SCORE2-OP) for those aged 70-75 for high CV risk countries, which include Poland (21). Predictors used in SCORE2/-OP are: age, sex, smoking, SBP, and non-HDL-C. We are aware SCORE2/-OP was developed to estimate risk in treatment-naïve persons, and that a significant portion of our subjects received lipid- and BP-lowering therapy. Nevertheless, we concluded applying this estimation tool along with FHS-ASCVD Risk calculation is of value.

### 2.3 Laboratory examinations

Blood was drawn between 8 and 10 a.m. after a fasting period of at least 8 hours from an antecubital vein, and used for regular examinations in the laboratory of our hospital apart from samples preserved for the determination of plasma Cts in all subjects, serum aldosterone and plasma direct renin concentration (DRC) in controls. These were centrifuged at 2,000 rpms for 20 minutes at 4 degrees C, aliquoted and stored at -80 degrees C until analysis.

Samples were analyzed in Central Clinical Laboratory in Gdańsk using standard laboratory methods (with a Siemens IMMULITE 1000 Immunoassay System for most biochemical tests, and an Abbott Architect analyzer, which applies the spectrophotometric method). Serum Cts was determined by an enzyme-linked immunosorbent assay (ELISA) by using a commercially-available diagnostic kit (SunRedBio, catalogue no: 201-12-8276; sensitivity: 0.268 ng/mL; assay range: 0.3-90 ng/mL). Cts concentrations above 90 ng/ml (n=15) were extrapolated based on ELISA standard curve.

Serum Cts, creatinine, sodium, potassium, aldosterone, renin, lipid profile (TC, HDL-C, LDL-C, and TGL), UA, hs-CRP, 24-h urinary protein and albumin excretion were determined both in AI patients and controls. Morning serum cortisol, dehydroepiandrosterone sulphate (DHEA-S), overnight 1 mg-DST cortisol and 24-h urinary cortisol were determined in AI patients. In most (n=50) AI patients, 24-h urinary meta- and normetanephrine excretion was determined, in others it had been performed prior to hospitalization. Screening for primary hyperaldosteronism based on aldosterone-to-renin ratio (ADRR) was performed without modifying antihypertensive medications in both AI patients and controls; there were no study participants with both HT and an ADRR above 2 ng/dL:μIU/mL.

### 2.4 Ambulatory blood pressure monitoring

24h ABPM was conducted using a Spacelabs Ontrak 90227 monitor on the non-dominant arm. During the day BP was recorded every 20 minutes, while during nighttime rest every 30 minutes. ABPM was repeated or not considered in the analysis if more than 30% of measurements were invalid. Normal results were adopted according to the European Society of Cardiology/European Society of Hypertension 2018 guideline: <130/80 mmHg for the 24-h period, <135/85 mmHg for daytime, and <120/70 mmHg for nighttime (22). Patients were classified as ‘non-dippers’ if their mean diurnal SBP and DBP were not at least 10% higher than nocturnal (22).

### 2.5 Transthoracic echocardiography

All measurements were performed in accordance with the recommendations endorsed by the American Society of Echocardiography and the European Association of Cardiovascular Imaging (23). Three on-site cardiology consultants with an expertise in ultrasonography performed TTE using the GE Vivid E9/E95 ultrasound system.

Measurements included left-ventricular (LV) internal dimension in diastole (LVIDd) and systole (LVIDs), LV ejection fraction (LVEF) according to modified Simpson’s rule (24), posterior LV wall thickness (LVPWd), and interventricular septal thickness (IVSd). LV mass (LVM) was calculated with the cube formula: LVM (g)=0.8×1.04 ×[(LVEDd+IVSd+LVPWd)^3^−LVEDd^3^]+0.6. LVM was indexed to body surface area (BSA) calculated using the DuBois formula (BSA=0.007184×H^0.725^×W^0.425^): LVM index (LVMI)=LVM/BSA. Relative wall thickness (RWT) was calculated with the formula: RWT=(2×LVPWd)/LVEDd. Left ventricular hypertrophy (LVH) was defined as LVMI >95 g/m^2^ for females and >115 g/m^2^ for males. RWT was used to further classify LVH as either concentric (RWT >0.42) or eccentric (RWT ≤ 0.42).

Disk summation technique from apical four and two-chamber views was used to determine left atrial volume (LAV), which was indexed to BSA: LAV index (LAVI) (ml/m^2^) = LAV/BSA (15). Apical four-chamber view was used to record peak blood flow velocity from LV relaxation in early diastole (E) and peak velocity flow in late diastole (A). Since LVEF was normal in all study participants, four criteria were applied to assess diastolic function: (1) LAVI ≥34 ml/m^2^, (2) tricuspid regurgitation velocity (TR) ≥2.8 m/s, (3) ratio of E to average early mitral annular velocity (e’) ≥14, (4) septal e’<7 cm/s or lateral e’<10 cm/s. Indeterminate diastolic function was stated if two criteria were met, and dysfunction if three or four (15).

### 2.6 Common carotid artery USG

Maximum carotid intima-media thickness (CIMT) measurements were recorded using echo-tracking technology on the distal wall of the right carotid artery, 1 to 3 cm below the carotid artery bifurcation. The presence of atherosclerotic plaques (ASP) defined as a CIMT ≥1.5 mm, or by a focal increase in thickness of 0.5 mm or 50% of the surrounding CIMT value was also recorded.

### 2.7 Statistical analysis

Data were analyzed using R-studio. Discrete variables were presented as number (n) or n (percentage). Continuous quantitative data with a normal distribution were presented as mean ± standard deviation (SD), and in the case of a non-normal distribution as median (interquartile range, IQR). We used the Shapiro-Wilk test to determine if a data set was well-modeled by a normal distribution. To compare differences between two independent groups Welch’s t-test was used when variables were normally distributed or the Mann-Whitney U test in the case of non-normal distribution. One-way ANOVA and Tukey’s honestly significant difference (HSD) tests were used to compare three or more independent groups. Simple (bivariate) correlations were computed with the non-parametric Spearman rank-order method (correlation coefficient r is given). Associations between dichotomous categorical variables were examined with Fisher’s exact test, and Benjamini-Hochberg Method was applied to correct for multiple testing.

Multiple regression models were applied to adjust for differences in Cts concentrations depending on potential confounding variables including gender, age, BMI, smoking status, comorbidities (HT, DMt2, MetS), and medications (ACEI/ARB, CCB, BB, diuretics, statins, and PPIs). An exhaustive search method was used to select factors that had the strongest relationship with Cts, i.e.: 1) gender, presence of 2) MetS, 3) HT, therapy with 4) statins, and 5) PPIs. The final multivariate model had a R-squared of 0.1831. Out of five variables included in the model only the presence of MetS (ß=-30, p=0.005) was significantly and negatively associated with Cts. For dichotomous dependent variables (Cts halves, HT, DMt2, and MetS) binary logistic regression was used to adjust for gender, age, and BMI. Significance was set at 0.05.

## 3 Results

### 3.1 Comparison of examined parameters between AI patients and controls

To assess whether the presence of an AI affected Cts levels, verification of matching between AI patients and controls was undertaken. These groups did not differ in regard to age, sex, BMI, smoking status, and comorbidities (incidence of HT, DM t.2, ASP, and dyslipidemia), see **Table 1**. Concerning subjects with HT, the number of patients on mono-, dual- and triple-drug therapy (including betablockers) was also comparable (**Supplementary Table 1**).

**Table 1:** Clinical, laboratory, ABPM, echocardiographic, and CCA sonography parameters in AI patients and controls.

	Controls	AI patients			p	adjusted p		
		all	NFAI	MACS	Cont. vs. AI	Cont.vs. NFAI	Cont. vs. MACS	NFAI vs. MACS
n	24	64	50	14	–	–	–	–
F:M ratio	19:5	45:19	35:15	10:4	0.592	1	1	1
Age [years]	62.2 ± 7.4	60.9 ± 8.8	60.1 ± 8.8	63.7 ± 9.3	0.487	0.575	0.854	0.334
BMI [kg/m²]	27.9 ± 4.6	28.6 ± 4.1	28.8 ± 4.2	28.3 ± 3.9	0.48	0.685	0.968	0.911
Obesity [n (%)]	8 (33.33%)	24 (37.5%)	20 (40%)	4 (28.6%)	0.807	0.928	1	0.928
HT [n (%)]	10 (41.7%)	36 (56.3%)	27 (54%)	9 (64.3%)	0.327	0.555	0.555	0.555
DMt2 [n (%)]	1 (4.17%)	12 (18.8%)	11 (22%)	1 (7.1%)	0.103	0.266	1	0.411
MetS [n (%)	11 (45.8%)	36 (56.3%)	27 (54%)	9 (64.3%)	0.527	0.621	0.621	0.621
Smokers [n (%)]	24 (33.3%)	28 (43.8%)	20 (40%)	8 (57.1%)	0.521	0.619	0.543	0.543
SCORE2/-OP [%] *	8 (4.5-14)	9 (7-13)	8 (6-12)	14 (11-18)	0.31	0.978	**0.021**	**0.005**
FHS-ASCVD score [%]	5.9 (2.8-12)	10.1 (4.8-16.4)	9.3 (4.6-16.4)	12.5 (8.4-15.6)	0.085	0.338	0.253	0.813
Catestatin [ng/ml]	4.5 (3.5-28)	6.5 (4.9-37)	7.2 (5-101)	6.1 (5-7.8)	**0.048**	0.71	0.7	0.274
HDL-C [mg/dl]	58.7 ± 12.1	54.0 ± 14.7	52.7 ± 14	58.7 ± 17	0.13	0.196	1	0.33
LDL-C [mg/dl]	120 ± 36.6	129 ± 48.2	130 ± 49.1	129 ± 46.8	0.32	0.663	0.828	0.998
TC [mg/dl]	202 ± 42.4	211 ± 53.1	210 ± 53.4	213 ± 53.8	0.45	0.813	0.803	0.978
TGL [<150 mg/dl]	106 (96.8-126)	130 (87-162)	130 (88.5-160)	129 (86.2-162)	0.25	0.414	0.965	0.727
UA [2.5-7 mg/dl]	5.3 (4.8-5.8)	5.1 (4.2-6.1)	5.1 (4.4 -6.1)	4.8 (4.2-5.8)	0.97	0.87	0.983	0.817
hs-CRP [<5 mg/l]	1.4 (1.1-2.5)	1.2 (0.7 - 1.7)	1.2 (0.7-1.6)	1.3 (0.6-2)	0.14	0.811	0.443	0.155
DST cortisol [<50 nmol/L]	–	26.9 ± 34	11.3 ± 17	79.1 ± 22.3	–	–	–	**<0.001**
24h SBP [mmHg]	118 ± 8.4	121 ± 9.7	120 ± 9.3	118 ± 7.7	0.5	0.62	0.982	0.612
24h DBP [mmHg]	70.6 ± 5.9	71.4 ± 7.8	71.7 ± 8.1	70.3 ± 6.5	0.61	0.818	0. 994	0.82
Non-dipper status [n (%)]†	6 (40%)	9 (28.1%)	9 (37.5%)	0	0.442	0.61	0.447	0.46
IVSd [mm]	10 (9-10)	11 (10-12)	11 (10-12)	12 (10.2-12)	**0.003**	**0.045**	**0.006**	0.295
LVIDd [mm]	46.1 ± 4.1	44.8 ± 4.5	45.2 ± 4.7	43.5 ± 3.7	0.193	0.651	0.183	0.427
LVIDs [mm]	29.9 ± 3.3	27.3 ± 3.1	27.3 ± 3.2	27.2 ± 3	**0.002**	**0.005**	**0.036**	0.991
LVPWd [mm]	9 (8-9.5)	10 (9-11)	10 (9-11)	11 (10-11.8)	**0.007**	0.08	**0.009**	0.268
LVM [g]	149 ± 27.2	165 ± 41.8	164 ± 42	169 ± 42.6	**0.047**	0.307	0.289	0.887
LVMI [g/m^2^]	86.4 ± 19.2	84.7 ± 18.5	92.7 ± 21	77.9 ± 8.7	**0.006**	**0.037**	**0.023**	0.21
LVH [n (%)] #	1 (4.4%)	13 (20.3%)	7 (14%)	6 (42.9%)	0.101	0.421	**0.007**	**0.028**
LAVI [ml/m^2^]	24.6 (15.1-31.9)	24.9 (15.9-31.6)	23.8 (13.1-31.8)	25.5 (20.5-30)	0.29	0.639	0.498	0.864
CIMT max [mm]	0.8 (0.7 -0.8)	1 (0.9 - 1.1)	1 (0.9 - 1.1)	0.9 (0.9 - 1)	< **0.01**	**<0.01**	**0.007**	0.996
ASP [n (%)]	2 (9.5%)	19 (29.7%)	16 (32%)	3 (21.4%)	0.117	0.215	0.526	0.526

Data are presented as number, n, (percentage, %), mean ± standard deviation or median (interquartile range) depending on distribution; p values were adjusted for multiple comparisons with Benjamini-Hochberg adjustment (for qualitative variables) and TukeyHSD test (for quantitative variables); bold font denotes significant (<0.05) p values; *only nondiabetic patients were included in SCORE2/-OP risk estimation (n= 23, 52, 39, and 13, respectively for Cont., AI, NFAI and MACS patients); ^#^LVH was defined as values of LVMI exceeding 95 or 115 g/m2 in females and males respectively; † dipper status was considered only in patients with HT. ASP, atherosclerotic plaques; BMI, body mass index; CIMT, carotid intima media thickness; con., controls; DBP, diastolic blood pressure; DMt2, diabetes mellitus t2; DST, dexamethasone suppression test; FHS-ASCVD Risk – 10-year atherosclerotic cardiovascular disease risk calculated based on the Framingham Heart Study; HDL-C, high density lipoprotein cholesterol; HT, hypertension; hs-CRP, high sensitivity C-reactive protein; IVSd, interventricular septal end diastole; LAVI, left atrial volume index; LDL-C, low density lipoprotein cholesterol; LVH, left ventricular hypertrophy; LVIDd, left ventricular internal diameter end-diastole; LVIDs, left ventricular internal diameter end systole; LVM, left ventricular mass; LVMI, LVM index; LVPWd, left ventricular posterior wall end diastole; MetS, metabolic syndrome; SBP, systolic blood pressure; SCORE2/-OP, Systematic Coronary Risk Estimation 2/-Older People; TC, total cholesterol; TGL, triglycerides, UA, uric acid.

Among AI patients, there were 14 with MACS and 31 classified as NFAI; analyses for AI patients and these two subgroups were performed separately.

Ten-year FHS-ASCVD Risk was comparable between patients with an AI/NFAI/MACS and controls, while in nondiabetic subjects, SCORE2/-OP was significantly higher in patients with MACS than in controls and patients with NFAI: 14% (11-18) vs. 8% (4.5-14), p = 0.021, and 8% (6-12), p = 0.005, respectively (**Table 1**). Still, this CVD risk index was comparable between controls and all AI patients (p=0.31), which illustrates effective matching between these groups.

**Figure 1:**
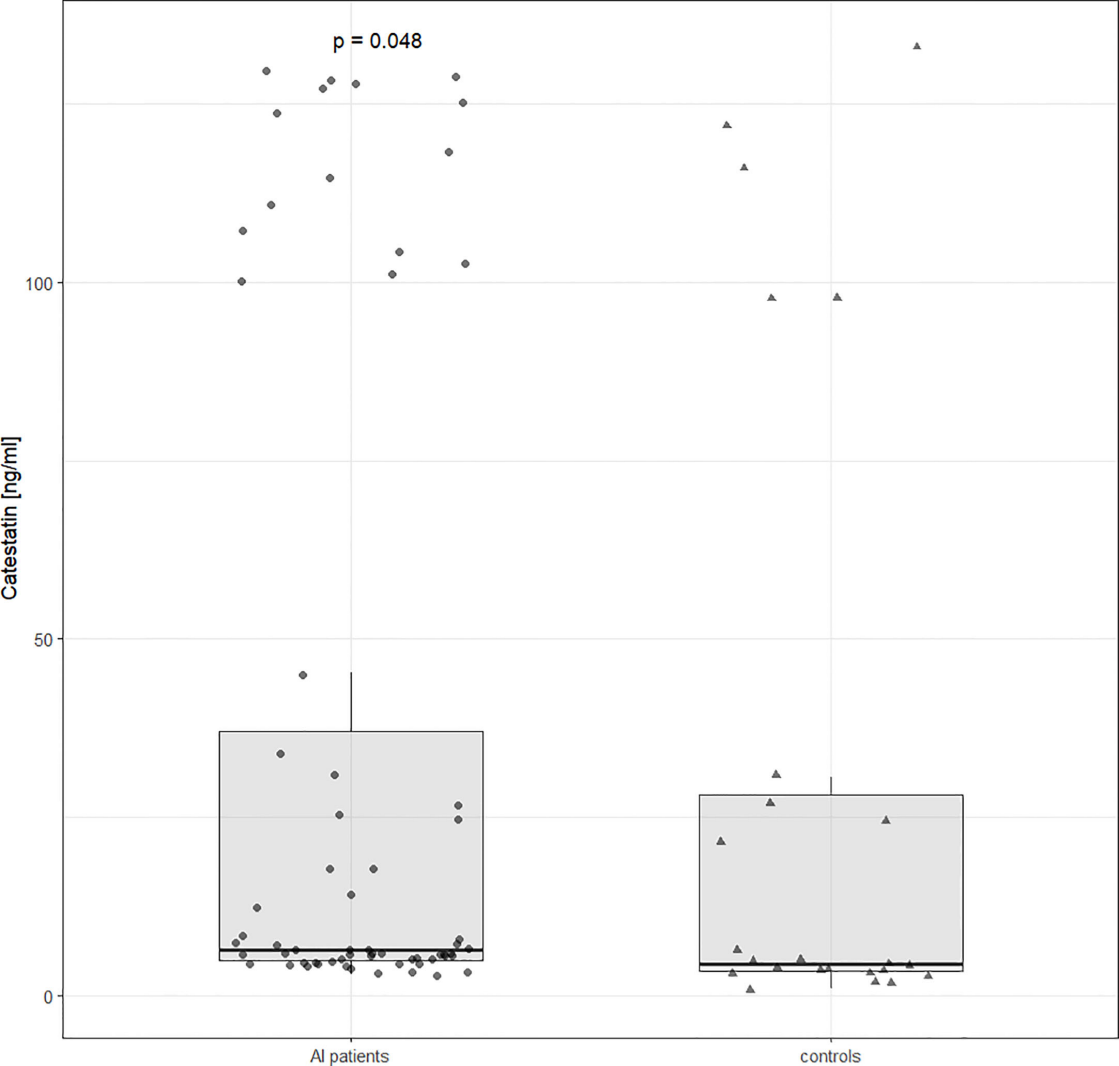
Catestatin distribution in controls and AI patients. Boxplot and data distribution with dots (AI patients) and triangles (controls) indicating individual datapoints. Unadjusted p value was determined using the Mann Whitney U test. AI, adrenal incidentaloma.

Cts distribution was bimodal both in AI patients and controls. Unadjusted Cts was slightly higher in AI patients: 6.45 (4.9-37) vs. 4.5 (3.5-28) ng/ml, p=0.047 (**Figure 1**). However, after adjusting for potential confounding variables (gender, age, and BMI), solely BMI and male gender were significantly (negatively) associated with Cts (ß=-28.3, p=0.01 and ß=-2.3, p=0.04, respectively) but not the presence of an AI (ß=-8.1, p=0.44).

Lipid profile, hs-CRP, as well as UA were comparable between controls and AI patients, be it with a NFAI or MACS. Proteinuria and albuminuria were normal in all study participants (respectively below 150 and 30 mg/24h). ABPM parameters (SBP, DBP, and pulse rate, PR) were comparable between AI patients and controls (**Table 1** and **Supplementary Table 1**).

Concerning TTE, there were significant differences in IVSd, LVPWd, and LVMI between AI patients and controls (respectively 11 (10-12) vs. 10 (9-10) mm, p=0.003; 10 (9-11) vs. 9 (8-9.5) mm, p=0.007; 86.4 ± 119.2 vs. 84.7 ± 18.5 g/m^2^, p=0.001). Moreover, LVH was more prevalent in MACS patients than controls (42.9% vs. 4.4%, p=0.007) and NFAI patients (42.9% vs. 14%, p=0.028).

Maximum CIMT was higher in patients with an AI, be it with a NFAI or MACS, than in controls: 1 (0.9-1.1) vs. 0.8 (0.8-0.9) mm, p<0.01. However, there were no differences in maximum CIMT between patients with a NFAI and MACS (**Table 1**). A trend toward a higher prevalence of an ASP in AI, NFAI, and MACS patients (29.7%, 32%, 21.43%, respectively) than controls (9.5%) could be observed (p=0.12) (**Table 1**).

### 3.2 Catestatin in clinically-specified patient groups

**Figure 2:**
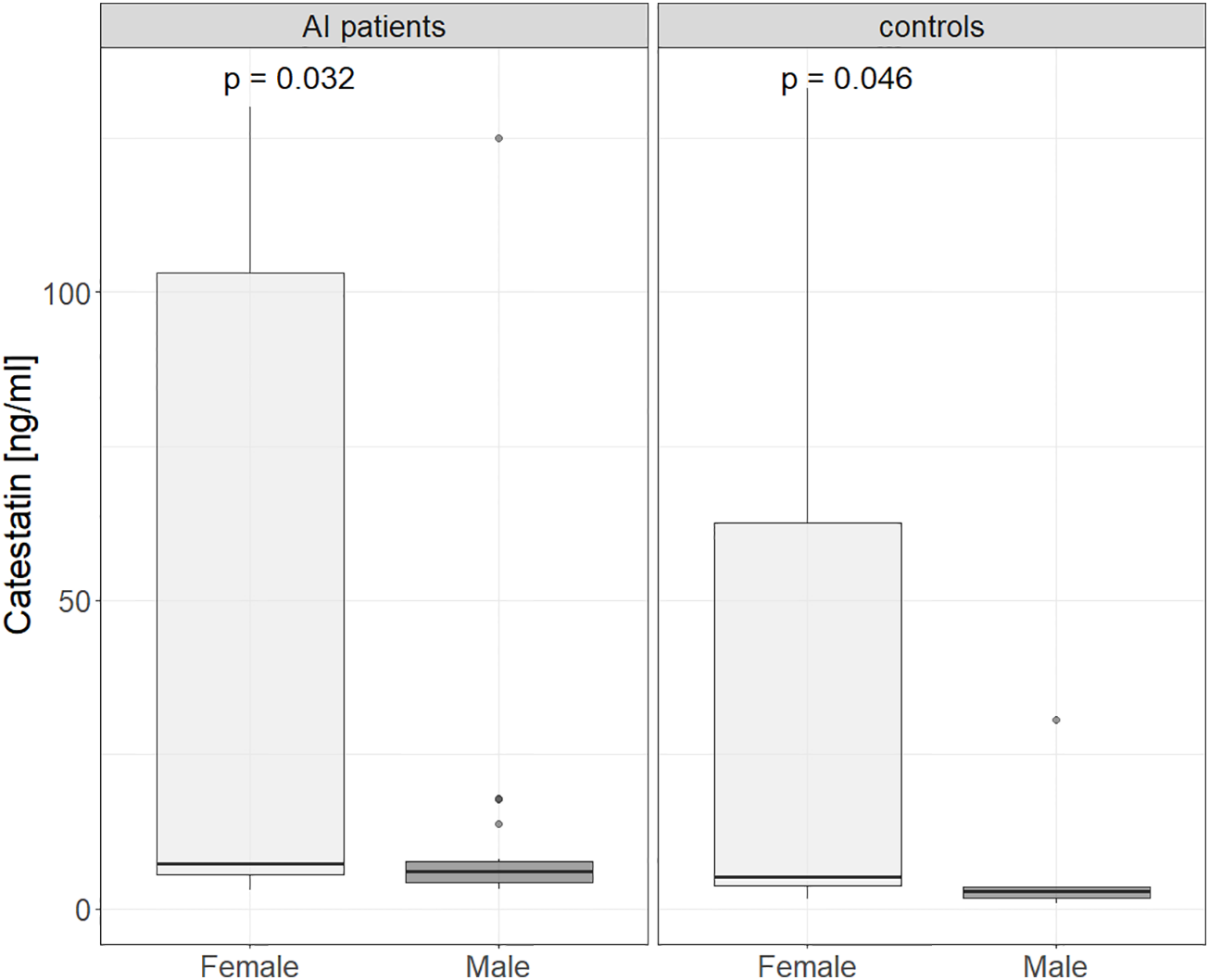
Catestatin in male and female controls and AI patients. Boxplot chart. P-value was determined using the Mann-Whitney U test. AI, adrenal incidentaloma.

Upon comparing Cts levels between controls with normal adrenal morphology and AI patients, peptide’s levels were tested in different patient groups. Cts was higher in women than in men: 7 (4.8-100) vs. 4.9 (4-7.4) ng/ml, p=0.015, and the difference between sexes was significant in both AI patients (7.3 (5.5-103) vs. 6 (4.26-7.6) ng/ml, p=0.03) and controls (5.1 (3.8 - 62.6) vs. 2.8 (1.7 - 3.5) ng/ml, p = 0.043), see **Figure 2**.

**Figure 3:**
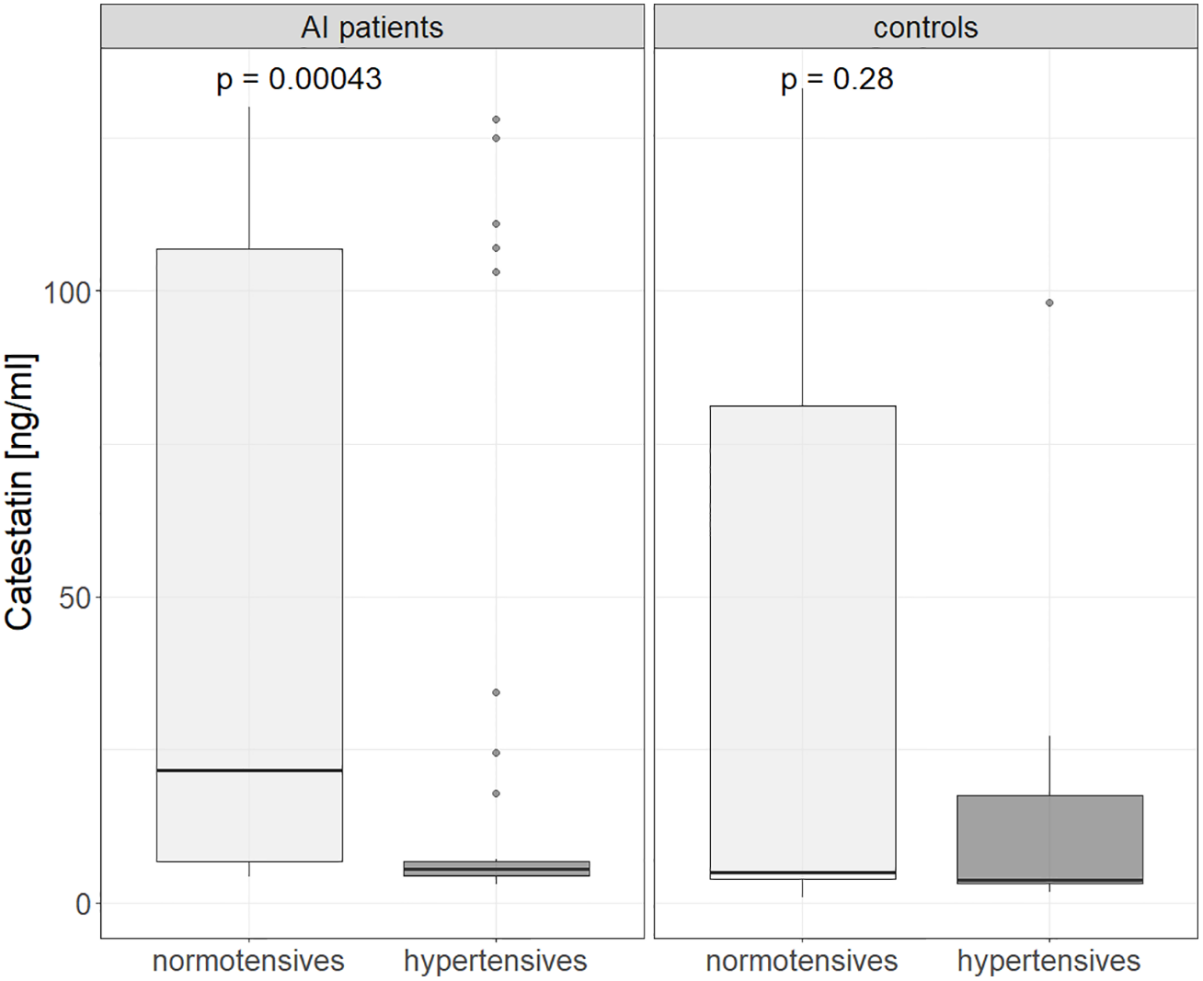
Catestatin in normotensive and hypertensive in controls and AI patients. Boxplot chart. P-values were determined using the Mann-Whitney U test. AI, adrenal incidentaloma.

**Figure 4:**
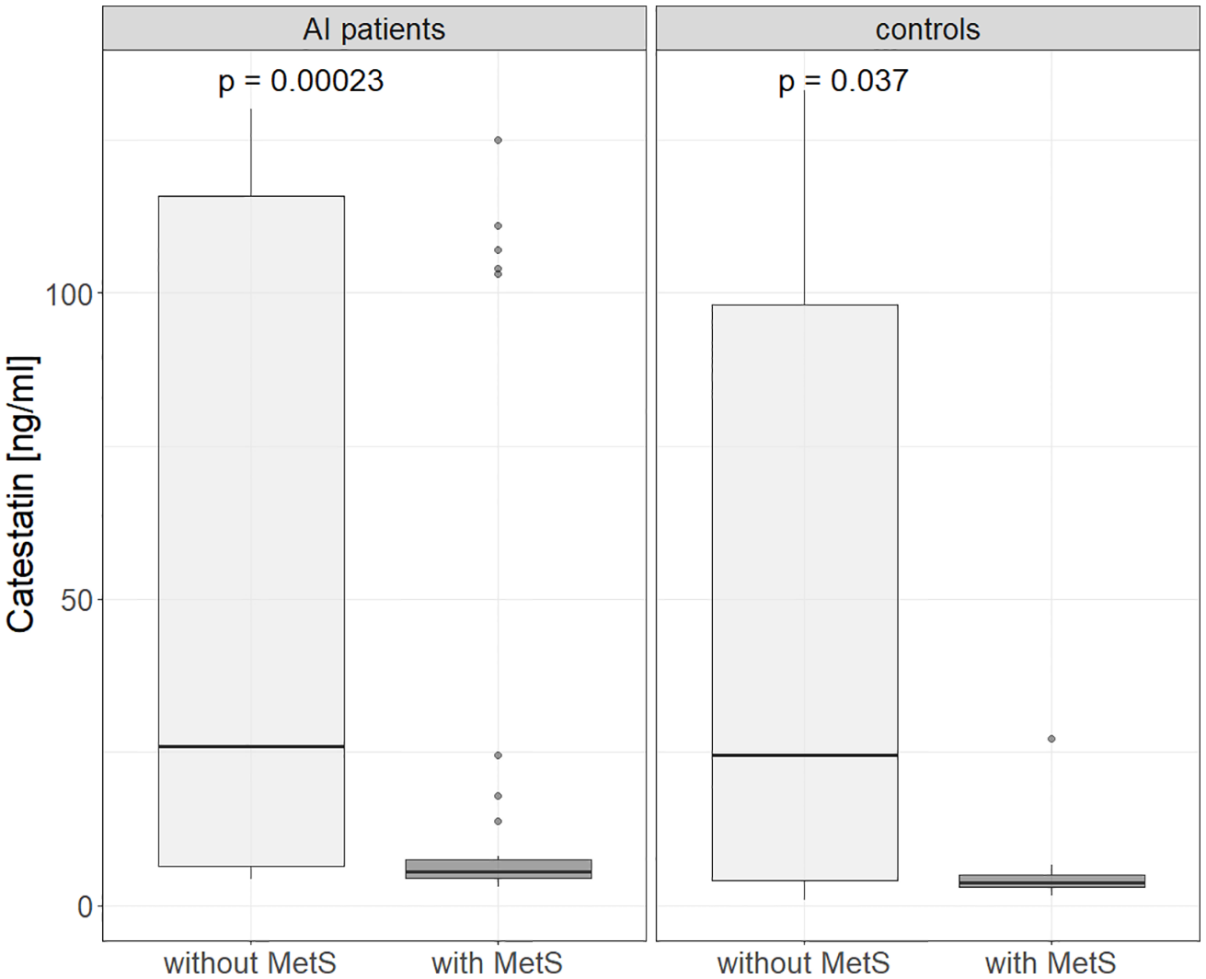
Catestatin in subjects without and with metabolic syndrome in controls and AI patients. AI, adrenal incidentaloma; MetS, metabolic syndrome. P-values determined using the Mann-Whitney U test.

Further, in AI patients and controls analyzed together Cts was lower in hyper- versus normotensive subjects: 5.6 (4-7.1) vs. 15.8 (5.2-103) ng/ml, p=0.003, which was also found for AI patients alone: 5.6 (4.36-6.82) vs. 21.7 (6.85-107), p < 0.001) (**Figure 3**). Cts was also significantly lower in subjects with MetsS than in those without it: 25.7 (5.8-115) vs. 5.2 (3.9- 6.9) ng/ml, p<0.01 (**Figure 4**), regardless of potential confounders (gender, age, BMI, presence of an AI and/or HT, statin and PPI use). We confirmed these differences (normo- versus hypertensive subjects as well as those without and with MetS) in women but not men (probably due to their low number). Cts in hypertensive AI females was lower than in normotensive ones: 5.6 (4.7 – 11.6) vs. 45.2 (8.2 – 118) ng/ml, p <0.01, and also lower in those with MetS than without it, both among AI patients: 5.6 (4.8-6.9) vs. 34.3 (7.7 - 121) ng/ml, p= <0.01 and controls: 3.8 (3.7-5.1) vs. 61.2 (8.4-112) ng/ml, p = 0.025 (**Supplementary Figures 1**, **2**).

There were no differences in Cts between obese and non-obese subjects, smokers and non-smokers, or, among subjects with HT, ‘dippers’ and ‘non-dippers’.

### 3.3 Correlations between catestatin and laboratory, TTE, and CCA USG parameters

**Figure 5:**
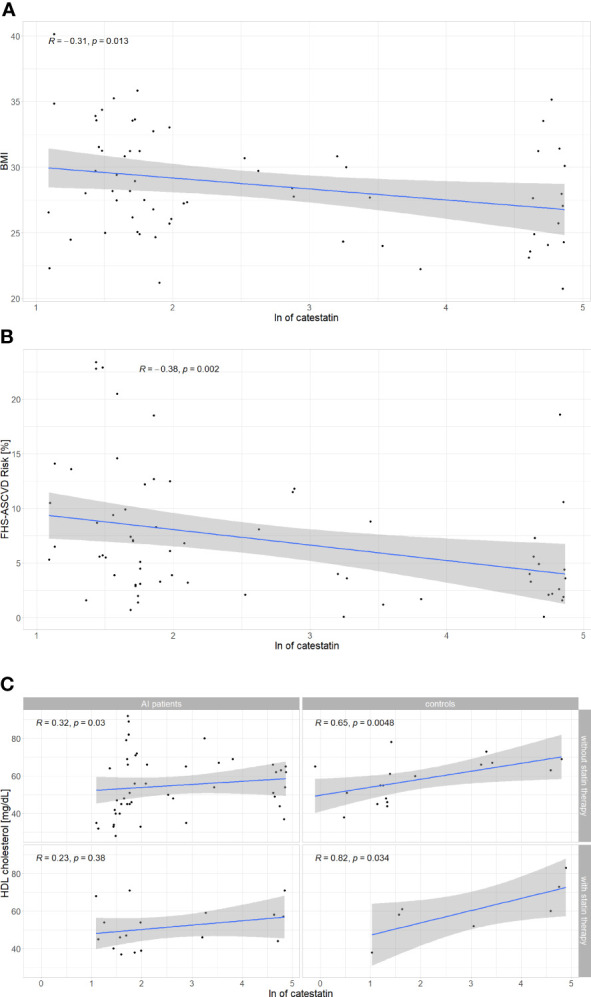
Correlations between catestatin and: **(A)** BMI in AI patients; **(B)** FHS-ASCVD Risk in AI patients; **(C)** HDL-C in AI patients and controls. Correlations were computed by the Spearman rank-order method. For HDL-C, correlations were tested separately in subjects with and without statin therapy. AI, adrenal incidentaloma, BMI, body mass index, FHS-ASCVD Risk - 10-year atherosclerotic cardiovascular disease risk calculated based on the Framingham Heart Study; HDL-C, high density lipoprotein cholesterol, ln, natural logarithm.

To further investigate associations between Cts and CVD risk, correlations were tested between peptide’s levels and other parameters. In AI patients, weak correlations were found between Cts and: BMI (r=-0.31) (**Figure 5A**), FHS-ASCVD Risk (r=-0.42) (**Figure 5B**), and HDL-C (r=0.32) regardless of statin therapy (**Figure 5C**). Interestingly, among participants without it, there were also positive correlations between Cts and: TC and LDL-C (r=0.36 for both) (**Table 2** and **Supplementary Figures 3**, **4**). In AI patients and controls analyzed as a whole a negative correlation between Cts and UA was also observed (r=-0.27, p=0.01), while for each group analyzed separately, significance has not been reached probably due to sample size.

**Table 2:** Correlations between catestatin and examined parameters.

Correlation between Cts and	Control group (n = 24)		AI patients (n = 64)		Both groups (n = 88)	
	r	p	r	p	r	p
Age	0.246	0.247	-0.142	0.262	-0.016	0.7
BMI	-0.293	0.164	**-0.308**	**0.013**	**-0.27**	**0.009**
HDL-C	**0.704**	< **0.001**	**0.306**	**0.014**	**0.344**	**0.001**
HDL-C *	**0.649**	**0.005**	**0.317**	**0.03**	**0.317**	**0.011**
LDL-C	0.102	0.634	0.15	0.236	0.153	0.15
LDL-C *	**0.573**	**0.016**	**0.361**	**0.003**	**0.361**	**0.003**
TC	0.215	0.313	0.118	0.353	0.16	0.137
TC *	**0.568**	**0.017**	**0.295**	**0.044**	**0.364**	**0.003**
TGL	-0.216	0.145	-0.19	0.131	-0.143	0.183
TGL *	-0.085	0.746	0.215	0.313	-0.142	0.263
UA $	-0.37	0.07	-0.209	0.1	**-0.27**	**0.01**
hs-CRP	0.191	0.407	0.005	0.97	-0.045	0.68
LVMI	0.149	0.488	-0.009	0.942	0.05	0.645
LAVI	0.13	0.545	-0.202	0.109	-0.121	0.263
Maximum CIMT	-0.09	0.697	-0.136	0.286	-0.03	0.774
SCORE2/-OP #	0.127	0.563	- 0.064	0.652	0.05	0.651
FHS-ASCVD Risk [%]	-0.237	0.265	**-0.42**	**< 0.001**	**-0.24**	**0.022**

Correlations were computed by the Spearman rank-order method; bold font denotes statistically significant correlations; *denotes scorrelations in participants without statin therapy: n=17 and 47, respectively for controls and AI patients; ^$^ patients with and without medications that could lower uric acid (allopurinol, n = 1) were analyzed separately and the results were the same; # denotes correlations in nondiabetics only: n= 23 and 52, respectively for controls and AI patients; BMI, body mass index; CIMT, carotid intima media thickness; FHS-CVD, 10-year atherosclerotic cardiovascular disease risk calculated based on the Framingham Heart Study; HDL-C, high density lipoprotein cholesterol; hs-CRP, high sensitivity C-reactive protein; LAVI, left atrial volume index; LDL-C, low density lipoprotein cholesterol; LVMI, left ventricular mass index; r, correlation coefficient; SCORE2/-OP, Systematic Coronary Risk Estimation 2/-Older People; TC, total cholesterol; TGL, triglycerides; UA, uric acid.

Analyses in subjects of each sex revealed Cts correlated with HDL (r=0.31, p=0.014), BMI (r=-0.29, p=0.019), and UA (r=-0.27, p=0.031) in women, but not in men (respectively r=0.13, p=0.53; r=-0.06, p=0.78; r=-0.12, p=0.56). A negative correlation between Cts and FHS-ASCVD Risk was also recorded in women with an AI (r=-0.3, p=0.049) but not in female controls (r =0.025, p=0.92), nor men with an AI (n=19, r=- 0.37, p=0.12). Correlations for male controls were not tested due to a low number of these subjects (n=5).

Concerning hormonal tests, only a weak correlation between Cts and DRC was observed, but not with aldosterone, nor ADRR (**Supplementary Table 2**). Cts did not correlate with ABPM, CCA USG, nor TTE parameters (LVPWd, IVSd, LVMI, LAVI) (**Table 2** and **Supplementary Table 2**).

### 3.4 Clinical, laboratory, and TTE parameters according to catestatin categories

Further analyses were performed among AI patients based on Cts categories. First, adjustment for gender, age and BMI in binary logistic regression analysis revealed AI patients in the lower half of Cts concentrations (median 6.5, IQR 4.9-37 ng/ml) compared to those in the upper had a higher prevalence of HT (OR 0.17, CI 0.05-5.37, p=0.003), and MetS (OR 0.21, CI 0.06-7.51, p=0.018). Moreover, BMI, 24-SBP, and FHS-ASCVD Risk were also higher in the former (respectively 30.1 ± 4 vs. 27.2 ± 3.6 kg/m^2^, p=0.004; 123 ± 7.4 vs. 117 ± 9.5 mmHg, p=0.022; 13.2%(8.9-19.2) vs. 6.3%(4.2-10.8), p=0.002), as summarized in **Table 3**.

**Table 3:** Clinical, laboratory, ABPM, echocardiographic, and CCA sonography parameters in AI patients according to catestatin category.

Cts half [ng/ml]	Lower (Cts < 6.5)		Upper (Cts ≥6.5)		p subgroups	p halves
Cts subgroup [ng/ml]	Very low (< 5)	Low (5 ≤ Cts < 6.5)	Intermediate (6.5 ≤ Cts ≤ 45.2)	High (Cts ≥ 100)		
n	17	15	17	15	**-**	**-**
F:M ratio	8:9	12:3	11:6	14:1 *	**0.027**	0.274
Age [years]	61.9 ± 9.4	61.7 ± 5.9	60.7 ± 11.5	59.2 ± 7.2	0.827	0.412
BMI [kg/m²]	30.4 ± 4.6	29.9 ± 3.4	27.1 ± 3.2	27.4 ± 4.2	**0.034**	**0.004**
Obesity [n (%)]	8 (47.1%)	8 (53.3%)	3 (17.7%)	5 (33.3%)	0.158	0.07
Smokers [n (%)]	7 (41.2%)	9 (40%)	5 (29.4%)	7 (46.7%)	0.378	0.45
PPI therapy [n (%)]	4 (23.5%)	4 (26.7%)	2 (11.8%)	3 (20%)	0.793	0.536
HT [n (%)]	14 (82.4%)	11 (73.3%)	6 (35.3%) *	5 (33.3%) *	**0.005**	**< 0.001**
>1 hypotensive drug [n(%)] †	7 (50%)	7 (63.6%)	3 (50%)	1 (20%)	0.495	0.470
DMt2 [n (%)]	6 (35.3%)	4 (26.7%)	1 (5.9%)	1 (6.7%)	0.09	**0.022**
MetS [n (%)]	14 (82.4%)	11 (73.3%)	6 (35.3%) *	5 (33.3%) *	**0.005**	**< 0.001**
Statin use [n (%)]	6 (35.3%)	3 (20%)	4 (23.5%)	4 (26.7%)	0.837	1
HDL-C [mg/dL]	42.9 ± 10.8	61.6 ± 17.8 *	56.7 ± 13.7 *	55.8 ± 9.5 *	**0.001**	0.076
LDL-C [mg/dL]	124 ± 49.4	121 ± 41.6	127 ± 36.3	146 ± 63.6	0.491	0.27
TC [mg/dL]	202 ± 52.6	206 ± 51	211 ± 41	225 ± 68.4	0.677	0.31
TGL [mg/dL]	143 (98 - 198)	133 (118 - 141)	121 (87 - 168)	105 (87.5 - 142)	0.159	0.36
Uric acid [mg/dL]	5.8 (4.9 - 6.8)	4.8 (4.1 - 5.8)	5.1 (4.2 - 6.1)	4.9 (4.3 - 5.8)	0.112	0.36
MACS [n (%)]	4 (23.5%)	4 (26.7%)	5 (29.4%)	1 (6.7%)	0.417	0.763
Hs-CRP [mg/L]	1.2 (0.8 - 3.9)	1.5 (1 - 2.1)	0.8 (0.6 - 1.5)	1.2 (0.8 - 2)	0.243	0.16
24h SBP [mmHg]	120.8 ± 7.8	124.7 ± 6.7	114.5 ± 8.5 #	119.2 ± 10.5	**0.01**	**0.009**
24h DBP [mmHg]	71.1 ± 9.6	74.9 ± 5.9	68.2 ± 6.1	72 ± 7.9	0.126	0.13
24h PR [bpm]	71.2 ± 9.8	72.9 ± 7.5	71.2 ± 6.5	72 ± 8.3	0.929	0.84
Non-dipper status [n (%)] †	4 (28.6%)	2 (18.2%)	1 (16.7%)	2 (40%)	0.723	0.685
LVMI [g/m^2^]	86.1 ± 18.1	84.2 ± 17	86.6 ± 19	88.9 ( ± 24.0)	0.931	0.61
LVH [n (%)]	1 (5.9%)	3 (20%)	2 (11.76%)	7 (46.7%)	**0.036**	0.213
LAVI [ml/m^2^]	26.6 ± 9.6	24 ± 8.6	24 ± 9.44	20.3 ± 7.1	0.258	0.2
Maximum CIMT [mm]	1 (0.9 - 1.2)	1 (0.9 - 1.1)	1 (0.9 - 1.1)	0.9 (0.9 -1.1)	0.685	0.393
ASP [n(%)]	7 (41.2%)	4 (26.7%)	4 (23.5%)	4 (26.7%)	0.709	0.585
SCORE2/-OP [%] ‡	8 (7 - 12)	12 (8 - 15.5)	7 (5 - 14.5)	8.5 (8 - 11.5)	0.572	0.29
FHS-ASCVD Risk [%]	14.3 (9.2 - 24.5)	11.2 (7.8 - 16.2)	7.1 (4.7 - 14.2)*	5.6 (3.8 - 10)*	**0.003**	**0.002**

AI patients were categorized based on catestatin concentrations into those in the lower and upper half, and further into four subgroups (two lowest were identical with first and second quartiles). Data are presented as number (percentage), mean ± standard deviation or median(interquartile range) depending on distribution; p-values were calculated with one-way ANOVA and Tukey’s HSD tests (quantitative variables) or Fisher’s exact test (categorical variables) with Benjamini-Hochberg correction for multiple testing. Bold font denotes significant (<0.05) p values; * denotes datapoints significantly different versus those for the ‘very low’ Cts subgroup in post hoc test; # denotes datapoints significantly different versus those for the ‘low Cts’ subgroup in post hoc test; † dipper status was considered only in patients with HT. ‡ Only nondiabetic patients were included in SCORE2/–OP risk estimation (n=11, 11, 16, and 14, for consecutive subgroups). ABPM, ambulatory blood pressure monitoring; ASP, atherosclerotic plaques; BMI, body mass index; bpm, beats per minute; CCA, common carotid artery; CIMT, carotid intima media thickness; DBP, diastolic blood pressure; DMt2, diabetes mellitus type 2; FHS-ASCVD Risk, 10–year atherosclerotic cardiovascular disease risk calculated based on data from the Framingham Heart Study; HDL-C, high density lipoprotein cholesterol; HT, hypertension; hs–CRP, high sensitivity C–reactive protein; LDL-C, low density lipoprotein cholesterol; LVH, left–ventricular hypertrophy; LVMI, left ventricular mass index; max, maximum; MACS, mild autonomous cortisol secretion; MetS, metabolic syndrome; PPI, proton pump inhibitor; PR, pulse rate; SCORE2/–OP, Systematic Coronary Risk Estimation 2/–Older People; SBP, systolic blood pressure; TC, Total cholesterol; TGL, triglycerides.

Second, based on Cts distribution, we divided AI participants into four subgroups, i.e. with: ‘very low’ (Cts <4.9 ng/ml, n=17), ‘low’ (≥4.9 and <6.5 ng/ml, n=15), ‘intermediate’ (≥6.5 and ≤45.2 ng/ml, n=17), and ‘high’ (≥100 ng/ml, n=15) Cts levels (**Table 3**). The first two comprised subjects from two lower quarters, while the ‘high Cts’ subgroup corresponded to almost all patients in the fourth quarter (15 instead of 16 patients were included since there were none in the 45.2 - 100 ng/ml range, see **Figure 1**).

These four Cts subgroups differed significantly in male-to-female ratio, prevalence of HT and MetS, mean/median BMI, HDL-C, 24h SBP, and FHS-ASCVD Risk (**Table 3**). *Post hoc* analysis revealed male gender was more prevalent in the ‘very low’ versus ‘high’ Cts subgroup (53% vs. 6.7%), while HT and MetS in the ‘very low’ versus ‘intermediate’ (82.4% vs. 35.3%, p=0.04 for both) and ‘high’ Cts subgroups (82.4% vs. 33.3%, p=0.04 for both). HDL-C was lower in the ‘very low’ than in the three remaining Cts subgroups (42.9 ± 42.9 vs. 61.6 ± 17.8, 56.7 ± 13.7, and 55.8 ± 9.5 mg/dL, adjusted p=0.001, 0.01, and 0.03, respectively). What is more, 24h SBP in the ‘low’ Cts subgroup was higher than in the ‘intermediate’ (124.7 ± 6.7 vs. 114.5 ± 8.5 mmHg, adj. p=0.008).

Ten-year FHS-ASCVD Risk in the ‘very low’ Cts subgroup was higher than in the ‘intermediate’ and ‘high’: 14.3% (19.2-24.5) vs. 7.1% (4.7-14.2) and 5.6% (3.8-9.8), respective adjusted p=0.014 and 0.005, in line with differences in gender proportions, prevalence of metabolic disorders and HT between the subgroups.

Fisher’s exact test revealed differences in LVH prevalence between four Cts subgroups, yet, without significance in pairwise comparisons with correction for multiple testing (Holm–Bonferroni method). No other significant differences were recorded between Cts halves and subgroups in TTE and CCA USG parameters (**Table 3**). Finally, clinical, laboratory and TTE parameters were also analyzed in the same Cts subgroups for females only (**Supplementary Table 3**). Significant differences were recorded for: HT and MetS prevalence, HDL-C and hs-CRP concentrations, mean 24h DBP, and FHS-ASCVD Risk. Calculations for male AI subjects were not performed due to their low number.

## 4 Discussion

Clinical research on Cts is scarce, even though it deserves attention due to its protective effects on the CV system demonstrated *in vitro* and *in vivo*. To our knowledge, our study is the first to examine Cts in patients with an AI, and to show lower Cts in adult patients with MetS than those without it, as well as correlations between Cts and FHS ASCVD risk index.

A thorough assessment was undertaken to investigate associations between Cts and CV risk factors. It must be highlighted that Cts changes dynamically in response to sympathetic nervous system activation in a negative feedback mechanism (25). Also, multiple diseases and drugs lead to CgA secretion, which may affect the concentration of its derivatives, including Cts. For these reasons, we excluded patients with established CVD, stage 3-5 chronic kidney disease, cancer, etc., and controlled the use of PPIs (10, 12, 26, 27). Limitations of our study include a small, heterogeneous patient sample, lack of CgA determination (Cts : CgA ratios may have provided further insights) and hormonal work-up in controls.

Since CgA is not expressed in the adrenocortical adenoma tissue, Cts levels are unlikely to differ between subjects with and without an adrenal adenoma (28, 29), which is what we found here for AI patients compared to age-, sex-, and BMI-matched controls after adjusting for confounding factors. Other researchers showed higher plasma CgA in patients with an adrenal adenoma than in subjects without one, which may underlie a slightly higher unadjusted Cts in our AI patients than controls (24, 25). What should be noticed is a similar distribution of Cts levels in controls and AI patients, including the proportion of subjects with high (>97 ng/ml) Cts: 21% in the former and 23% in the latter. This further suggests that the presence of an AI does not affect Cts. Regarding hormonal activity, we found no differences in Cts between patients with MACS and NFAI, nor correlations between Cts and UFC or 1-mg DST cortisol, possibly due to small sample size.

**Figure 6:**
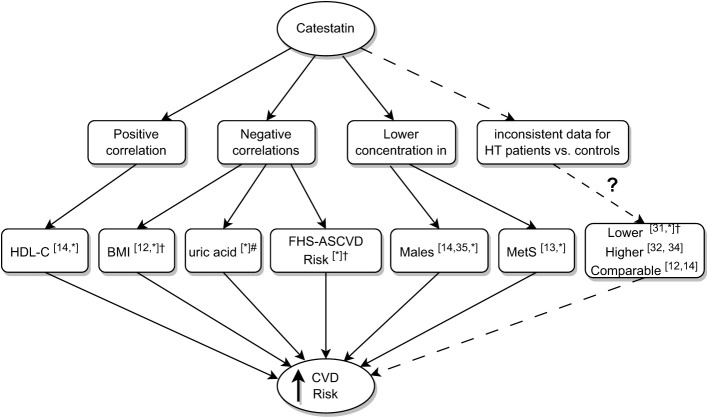
Summary of research on associations between low catestatin and cardiovascular risk. Superscript numbers indicate references to previous studies; *indicate results of the current study; ^#^correlation between uric acid and Cts did not reach significance in AI patients analyzed here, it did upon analyzing AI patients and controls together; ^†^significance achieved in AI patients. AI, adrenal incidentaloma, BMI, body mass index; Cts, catestatin; CVD, cardiovascular disease; FHS-ASCVD Risk, 10-year atherosclerotic cardiovascular disease risk calculated based on the Framingham Heart Study; HDL-C, high density lipoprotein cholesterol; HT, hypertension; MetS, Metabolic Syndrome.

Despite comparable age, BMI, male-to-female ratio, smoking status and comorbidities, IVSd, LVPWd, LVM, and LVMI were higher in AI patients than controls, which was driven by values recorded in subjects with MACS. Iacobellis et al. reported similar results (higher LVM in AI subjects versus controls, the difference depended on patients with MACS) (30). In our study, SCORE2/-OP was higher in MACS compared to NFAI patients and controls, which indicates increased CV risk associated with subclinical hypercortisolemia. The data support the hypothesis that chronic mild elevation of cortisol levels in AI patients adversely affect the CV system rather than the presence of an adrenal adenoma *per se*. Low Cts was associated here with a higher prevalence of male gender, HT, MetS, as well as BMI, 24h SBP, UA and lower HDL-C. Consequently, an association between Cts and ASCVD risk was recorded (**Figure 6**). In line with our results, Cts was lower in obese children with MetS than in those without it, and in normal-weight controls (13); O’Connor et al. showed Cts correlated negatively with BMI (12), and Durakoğlugil et al. reported a positive correlation between Cts and HDL-C (14). In the latter study, a negative correlation between plasma Cts and TGL concentration was also observed (14), which was not confirmed here. Surprisingly, among participants without statins, we recorded weak positive correlations between Cts and TC as well as LDL-C. The former may be connected with a positive correlation between Cts and HDL-C, however, the latter is difficult to explain, and requires further clarification.

To date, there are controversies regarding Cts in HT; lower Cts levels have been associated with this disease (12, 14, 31–35) (**Figure 6**). Here, Cts in AI subjects with HT was lower than in normotensive ones, however, the difference was not significant in the controls and we recorded no correlations between Cts and ABPM results. Our data add important facts to the discussion: low Cts levels are more common in HT, however, some patients do exhibit intermediate and high concentrations of the peptide. This was observed in individuals with effective hypotensive treatment revealed by 24-h monitoring.

Further, no significant associations were recorded here between Cts and TTE as well as CCA USG parameters. Small sample size clearly limits conclusions that can be drawn from these data. More sensitive methods (e.g. global longitudinal strain and microvasculature assessment) may have yielded different results.

Possibly, the most intriguing question is the clinical significance of high versus very low/low Cts in individuals with similar established CV risk factors. For instance, non-smoking females aged ca. 60, with overweight and HT (FHS-ASCVD Risk between 10 and 20%) were recorded both in the first half and highest quarter of Cts levels among AI patients. Longitudinal assessment of much larger populations is required to determine whether Cts provides protection against CVD. If so, determining therapeutic strategies that stimulate Cts would be beneficial.

## 5 Conclusions

We are the first to report that among persons without overt CVD other than primary HT, plasma Cts concentrations in patients with an AI are comparable to those of matched controls with normal adrenal morphology. Correlations between Cts and: HDL-C (positive) as well as BMI, UA and FHS-ASCVD Risk (negative) point at cardioprotective effects of the peptide. Data from ABPM, TTE and CCA intima-media assessment did not yield associations between Cts and BP or HT-mediated organ damage. It must be highlighted that many factors influence Cts, and further research is necessary to apply it as a biomarker.

## Data availability statement

The original contributions presented in the study are included in the article/**Supplementary Material**. Further inquiries can be directed to the corresponding author.

## Ethics statement

The studies involving human participants were reviewed and approved by the Independent Bioethics Committee for Scientific Research at Medical University of Gdańsk (NKBB/659/2019). The patients/participants provided their written informed consent to participate in this study.

## Author contributions

EZ secured ethical approval for the study, analyzed the data, and reviewed the literature. EZ and PK collected the data and wrote the manuscript. JS and AK performed echocardiography and ultrasound examination of the common carotid artery. PK and KS carried out critical interpretations. All authors contributed to the article, approved the submitted version, and are accountable for the content of the work.

## Supplementary material

Supplementary Figure 1 Catestatin in normotensive and hypertensive AI patients and controls - analyses in subjects of each sex. Boxplot chart. P-values determined using the Mann-Whitney U test. AI – adrenal incidentaloma.

Supplementary Figure 2 Catestatin in subjects without and with metabolic syndrome in controls and AI patients - analyses in subjects of each sex. Boxplot chart. P-values determined using the Mann-Whitney U test. AI – adrenal incidentaloma; MetS – metabolic syndrome.

Supplementary Figure 3 Correlations between catestatin and LDL-C level in AI patients and controls. Correlations were computed by the Spearman rank-order method and were tested separately in subjects with and without statin therapy. AI – adrenal incidentaloma, LDL-C – low density lipoprotein cholesterol, ln – natural logarithm.

Supplementary Figure 4 Correlations between catestatin and total cholesterol level in AI patients and controls. Correlations were computed by the Spearman rank-order method and were tested separately in subjects with and without statin therapy. AI – adrenal incidentaloma, ln – natural logarithm.
