# Supplementary material for: Low catestatin as a risk factor for cardiovascular disease – assessment in patients with adrenal incidentalomas
Source: Front Endocrinol (Lausanne). 2023 Jul 14;14:1198911. doi: 10.3389/fendo.2023.1198911 (PMC10379641; doi:10.3389/fendo.2023.1198911)
Supplement: Supplementary file 1 [file Table_1.docx]

Supplementary Material

Low catestatin levels as a cardiovascular risk factor - assessment in patients with incidentally discovered adrenal adenomas

**Ewa Zalewska*, Piotr Kmieć, Jakub Sobolewski, Andrzej Koprowski, Krzysztof Sworczak**

*** Correspondence:** Piotr Kmieć, piotrkmiec@gumed.edu.pl

17 Mariana Smoluchowskiego Street, 80214 Gdańsk, Poland, phone: +48 58 584 4809

# Supplementary Table 1. Additional clinical, laboratory, 24 h ambulatory blood pressure monitoring, and echocardiographic parameters in AI patients and controls.

|  | Controls | AI patients | | | p | adjusted p | | |
| --- | --- | --- | --- | --- | --- | --- | --- | --- |
|  |  | All | NFAI | MACS | Cont. vs  AI | Cont. vs NFAI | Cont. vs MACS | NFAI vs MACS |
| Additional clinical data | | | | | | | | |
| HT therapy [n (%)] | 10 (41.67 %) | 36 (56.25 %) | 27 (54 %) | 9 (64.29 %) | 0.327 | 0.555 | 0.555 | 0.555 |
| Beta-blockers [n (%)] * | 5 (50 %) | 20 (55.56 %) | 18 (66.67 %) | 2 (22.22 %) | 1 | 0.454 | 0.454 | 0.147 |
| ACEI/ARB [n (%)] * | 6 (60 %) | 21 (58.33 %) | 14 (51.85 %) | 7 (77.78 %) | 1 | 0.725 | 0.725 | 0.725 |
| Ca-blockers [n (%)] * | 2 (20 %) | 8 (22.22 %) | 5 (18.52 %) | 3 (33.33 %) | 1 | 1 | 0.942 | 0.942 |
| Diuretics [n (%)] * | 1 (10 %) | 10 (27.78 %) | 7 (25.93 %) | 3 (33.33 %) | 0.41 | 0.606 | 0.606 | 0.608 |
| Statin therapy [n (%)] | 7 (29.17 %) | 17 (26.56 %) | 14 (28 %) | 3 (21.23%) | 0.794 | 1 | 1 | 1 |
| AD [n (%)] # | 0 | 6 (12.77 %) | 5 (13.89 %) | 1 (9.09 %) | 0.182 | 0.489 | 0.59 | 1 |
| LDL-C ≥ 115 [n (%)] # | 13 (76.47 %) | 32 (68.08 %) | 24 (66.67 %) | 8 (72.73 %) | 0.758 | 1 | 1 | 1 |
| Low HDL-C † [n (%)] # | 2 (11.76 %) | 9 (19.15 %) | 11 (30.56 %) | 2 (18.18 %) | 0.485 | 0.469 | 1 | 1 |
| TGL≥150mg/dL [n(%)] # | 3 (17.64 %) | 14 (29.79 %) | 10 (27.78 %) | 4 (36.36 %) | 0.523 | 0.71 | 0.71 | 0.71 |
| PPI therapy [n (%)] | 7 (29.17 %) | 13 (20.31 %) | 9 (18 %) | 4 (28.57 %) | 0.401 | 0.686 | 1 | 0.686 |
| Additional laboratory results | | | | | | | | |
| Sodium [136-145 mmol/l] | 142 (140.5 – 143) | 140 (138 – 141) | 139.5 (138 - 141) | 140 (139 – 140.8) | **< 0.01** | **< 0.01** | **0.002** | 0.975 |
| Potassium [3.5-5.1 mmol/l] | 4.3 (± 0.4) | 4.33 (± 0.39) | 4.35 (± 0.34) | 4.25 (± 0.55) | 0.51 | 0.892 | 0.915 | 0.685 |
| Creatinine [<1.1 mg/dl] | 0.81 (± 0.15) | 0.78 (± 0.14) | 0.8 (±1.39) | 0.79 (±1.23) | 0.91 | 1 | 0.792 | 0.761 |
| eGFR [>60 ml/min/1.73 m^2^] | 97.2 (91.2 - 100) | 97.6 (91.1 - 101.5) | 98.1 (92.3 - 97.7) | 96.3 (89.9 - 101.3) | 0.56 | 0.547 | 0.986 | 0.774 |
| 24 h UFC [11.8-485.6 nmol/d] | - | 184.7 (94.9 - 314) | 184 (89.9 – 312) | 206 (140 – 338) | - | - | - | 0.41 |
| DHEA-S [0.35 - 8.37 ug/dL] $ | - | 69.25 (42.27 - 128.5) | 70.8 (43.2 – 128) | 64.1 (33.4 – 128) | - | - | - | 0.75 |
| Aldosterone [2.5-39.2 ng/dl] ‡ | 12.4 (10.5 - 16.7) | 7. (5.3 - 11.4) | 7.3 (5.3 – 11.3) | 8.9 (5.5 – 12.4) | **< 0.01** | 0.6 | 0.388 | 0.067 |
| Renin [4.4-46.1 μIU/ml] ‡ | 15.3 (4.3 - 39.3) | 11.5 (5.6 - 24.9) | 9.3 (5.7 - 21.2) | 19.2 (3.8 - 26.7) | 0.59 | 0.707 | 1 | 0.8 |
| ADRR [<2 ng/dL/mIU/L] | 0.91 (0.43 - 2.31) | 0.81 (0.45 - 1.41) | 0.8 (0.39 – 1.3) | 0.73 (0.5 – 1.94) | 0.4 | 0.07 | 0.505 | 0.821 |
| Additional 24 h ambulatory blood pressure monitoring data | | | | | | | | |
| 24h PR [mmHg] | 70.5 (± 7.64) | 71.8 (± 7.95) | 71.3 (± 8.16) | 73.3 (± 7.25) | 0.49 | 0.906 | 0.55 | 0.704 |
| SBP daytime [mmHg] | 123 (± 9.31) | 123 (± 9.12) | 124 (± 9.56) | 122 (± 7.37) | 0.834 | 0.905 | 0.905 | 0.699 |
| DBP daytime [mmHg] | 74.2 (± 4.78) | 74.6 (± 8.11) | 74.9 (± 8.48) | 73.5 (± 6.81) | 0.763 | 0.915 | 0.964 | 0.817 |
| PR daytime [mmHg] | 73.4 (± 8.18) | 73.9 (± 8.45) | 73.3 (± 8.68) | 75.7 (± 7.62) | 0.805 | 1 | 0.703 | 0.65 |
| SBP nighttime [mmHg] | 108 (± 11.1) | 108 (± 9.33) | 109 (± 9.51) | 104 (± 8.25) | 0.983 | 0.919 | 0.627 | 0.385 |
| DBP nighttime [mmHg] | 62.0 (± 7.76) | 61.7 (± 8.31) | 62.3 (± 8.70) | 59.5 (± 6.58) | 0.845 | 0.992 | 0.632 | 0.517 |
| PR nighttime [mmHg] | 62.9 (± 8.03) | 64.7 (± 7.29) | 64.5 (± 7.32) | 65.7 (± 7.41) | 0.339 | 0.691 | 0.542 | 0.874 |
| Additional echocardiography data | | | | | | | | |
| RWT [0.32-0.42] | 0.4 (0.3 – 0.4) | 0.45 (0.4 – 0.5) | 0.44 (0.38 - 0.5) | 0.49 (0.43 - 0.53) | **0.004** | 0.06 | **0.003** | 0.158 |
| LVM [g] | 149 (± 27.2) | 165 (± 41.8) | 164 (± 42.0) | 169 (± 42.6) | **0.047** | 0.307 | 0.289 | 0.887 |
| LVH [n (%)]⁂  Concentric [n]  Eccentric [n] | 1 (4.35%)  -  1 | 13 (20.31 %)  11  2 | 7 (14 %)  6  1 | 6 (42.86%)  5  1 | 0.101  **-**  **-** | 0.421  **-**  **-** | **0.007**  **-**  **-** | **0.028**  **-**  **-** |
| LVEF [>50%] | 64.5 (61.5 – 67) | 66 (63 – 69) | 67 (63 – 69) | 64 (63 – 67) | **0.045** | **0.03** | 0.804 | 0.345 |
| E/A [0.8-2] | 0.9 (0.74 - 1) | 0.86 (0.75 - 1) | 0.85 (0.73 - 1.04) | 0.9 (0.8 – 0.9) | 0.91 | 0.848 | 0.999 | 0.909 |

Legend: Data that is normally distributed is shown as the mean and standard deviation, and data that is not normally distributed is shown as the median and first and third quartiles. P-values were adjusted for multiple comparisons with a Benjamini & Hochberg adjustment (for qualitative variables) and Tukey HSD test (in case of quantitative variables). Bold font denotes statistically significant comparisons. * among hypertensive participants; # among participants without statin therapy (n= 17, 47, 36, and 11, respectively for Cont., AI, NFAI and MACS patients); † - < 40 mg/dL for men and < 45 mg/dL for women; $ referral ranges for female ages 50 to 59: 0.70 to 5.40 µmol/L, ages 60 to 69: 0.35 to 3.51 µmol/L, ages 69 and older: 0.46 to 2.43 µmol/L for male ages 50 to 59: 1.89 to 8.37 µmol/L, ages 60 to 69: 1.13 to 7.83 µmol/L, ages 69 and older: 0.76 to 4.72 µmol/L; ‡ - standards for subjects after activity; ⁂ - LVH was defined as values of LVMI exceeding 95 or 115 g/m^2^ in females and males respectively; AD – atherogenic dyslipidemia; ADRR - aldosterone/direct renin concentration ratio; con. – controls; DBP – diastolic blood pressure; DHEA-S - dehydroepiandrosterone sulfate; DST - Dexamethasone suppression test; E/A - early (E) to atrial (A) trans-mitral flow velocities ratio; F – female; HT – hypertension; PPI – proton pomp inhibitor; LVEF – left ventricular ejection fraction; LVH - left ventricular hypertrophy; N – norm; RWT - relative wall thickness; SBP – systolic blood pressure; UFC – urinary free cortisol.
