# Supplementary material for: Low catestatin as a risk factor for cardiovascular disease – assessment in patients with adrenal incidentalomas
Source: Front Endocrinol (Lausanne). 2023 Jul 14;14:1198911. doi: 10.3389/fendo.2023.1198911 (PMC10379641; doi:10.3389/fendo.2023.1198911)
Supplement: Supplementary file 2 [file Table_2.docx]

Supplementary Material

Low catestatin levels as a cardiovascular risk factor - assessment in patients with incidentally discovered adrenal adenomas

**Ewa Zalewska*, Piotr Kmieć, Jakub Sobolewski, Andrzej Koprowski, Krzysztof Sworczak**

*** Correspondence:** Piotr Kmieć, piotrkmiec@gumed.edu.pl

17 Mariana Smoluchowskiego Street, 80214 Gdańsk, Poland, phone: +48 58 584 4809

# Supplementary Table 2. Catestatin correlations with selected laboratory, 24 h ambulatory blood pressure monitoring, and echocardiographic parameters.

| Correlation of Cts with: | Control group (n = 24) | | Study group (n = 64) | | Both groups (n= 88) | |
| --- | --- | --- | --- | --- | --- | --- |
|  | r | p-value | r | p-value | r | p-value |
| 24 h UFC | - | - | 0.113 | 0.374 | - | - |
| 1 DST cortisol | - | - | -0.114 | 0.383 | - | - |
| DHEA-S | - | - | 0.04 | 0.751 | - | - |
| 24-h urinary NMN excretion (n = 50) | - | - | -0.009 | 0.952 | - | - |
| 24-h urinary MN excretion (n = 50) | - | - | 0.034 | 0.812 | - | - |
| 24-h urinary MTY excretion (n = 50) | - | - | 0.06 | 0.677 | - | - |
| Aldosterone | -0.07 | 0.737 | -0.04 | 0.687 | -0.04 | 0.687 |
| Renin | -0.154 | 0.474 | **-0.231** | **0.031** | **-0.231** | **0.031** |
| ADRR | 0.137 | 0.523 | 0.192 | 0.075 | 0.191 | 0.075 |
| 24h ABPM SBP | -0.03 | 0.887 | -0.143 | 0.283 | -0.066 | 0.556 |
| 24h ABPM DBP | -0.03 | 0.884 | -0.11 | 0.503 | -0.075 | 0.503 |
| 24h ABPM HR | 0.025 | 0.908 | -0.034 | 0.8 | -0.031 | 0.783 |
| IVSd | 0.005 | 0.98 | -0.176 | 0.163 | -0.044 | 0.685 |
| LVPWd | -0.089 | 0.68 | -0.067 | 0.599 | -0.015 | 0.886 |
| RWT | 0.07 | 0.744 | -0.072 | 0.572 | 0.051 | 0.635 |

Legend: Correlations were computed by the Spearman rank-order method. Bold font denotes statistically significant correlations. 1 DST - 1-mg overnight dexamethasone suppression test; ABPM – ambulatory blood pressure monitoring; ADRR - aldosterone/direct renin concentration ratio; DBP – diastolic blood pressure; DHEA-S - dehydroepiandrosterone sulfate; HR – heart rate; IVSd - interventricular septal end diastole; LVPWd - left ventricular posterior wall end diastole; r - correlation coefficient; MN – metanephrine; MTY – methoxytyramine; NMN – normetanephrine; RWT - relative wall thickness; SBP – systolic blood pressure; UFC – urinary free cortisol.
