# Supplementary material for: Low catestatin as a risk factor for cardiovascular disease – assessment in patients with adrenal incidentalomas
Source: Front Endocrinol (Lausanne). 2023 Jul 14;14:1198911. doi: 10.3389/fendo.2023.1198911 (PMC10379641; doi:10.3389/fendo.2023.1198911)
Supplement: Supplementary file 3 [file Table_3.docx]

Supplementary Material

Low catestatin levels as a cardiovascular risk factor - assessment in patients with incidentally discovered adrenal adenomas

**Ewa Zalewska*, Piotr Kmieć, Jakub Sobolewski, Andrzej Koprowski, Krzysztof Sworczak**

*** Correspondence:** Piotr Kmieć, piotrkmiec@gumed.edu.pl

17 Mariana Smoluchowskiego Street, 80214 Gdańsk, Poland, phone: +48 58 584 4809

**Supplementary Table 3.** Clinical, laboratory, ABPM, echocardiographic, and CCA sonography parameters in AI female patients according to catestatin category.

| Cts half [ng/ml] | Lower (Cts < 6.5) | | Upper (Cts ≥6.5) | | p  subgroups | p  halves |
| --- | --- | --- | --- | --- | --- | --- |
| Cts subgroup [ng/ml] | Very low  (< 5) | Low  (5 ≤ Cts < 6.5) | Intermediate  (6.5 ≤ Cts < 45.2) | High  (Cts ≥ 100) |  |  |
| n | 8 | 12 | 11 | 14 | **-** | **-** |
| Age [years] | 65.5 ± 5.53 | 60.7 ± 5.42 | 63.2 ± 10.7 | 58.8 ± 7.30 | 0.213 | 0.403 |
| BMI [kg/m²] | 30.0 ± 6.01 | 30.1 ± 3.5 | 26.4 ± 3.30 | 27.1 ± 4.16 | 0.09 | **0.0134** |
| Obesity [n (%)] | 3 (37.5 %) | 7 (58.3 %) | 2 (18.2 %) | 4 (28.6%) | 0.239 | 0.116 |
| Smokers [n (%)] | 3 (37.5 %) | 7 (58.3 %) | 4 (36.4%) | 7 (50 %) | 0.705 | 0.769 |
| PPI therapy [n (%)] | 2 (25 %) | 3 (25 %) | 2 (18.2 %) | 3 (21.43 %) | 1 | 0.731 |
| HT [n (%)] | 8 (100 %) | 7 (58.3 %) | 4 (36.4%)* | 4 (28.6%)* | **0.004** | **0.002** |
| >1 hypotensive drug [n(%)] † | 4 (50%) | 5 (41.7 %) | 3 (27.3 %) | 1 (7.5 %) | 0.096 | **0.049** |
| DMt2 [n (%)] | 1 (12.5 %) | 4 (33.3 %) | 0 | 1 (7.5 %) | 0.113 | 0.074 |
| MetS [n (%)] | 7 (87.5 %) | 9 (75 %) | 2 (18.2 %) ***** | 4 (28.6%) | **0.002** | **< 0.001** |
| Statin use [n (%)] | 3 (37.5 %) | 2 (16.7 %) | 4 (36.4%) | 4 (28.6 %) | 0.705 | 0.745 |
| HDL-C [mg/dL] | 48.1 ± 11.8 | 65.3 ± 17.7 * | 59.6 ± 12.3 | 57.1 ± 8.24 | **0.045** | **0.013** |
| LDL-C [mg/dL] | 126 ± 40.6 | 128 ± 41.6 | 117 ± 34.3 | 147 ± 65.8 | 0.468 | 0.625 |
| TC [mg/dL] | 198 ± 41.8 | 217 ± 47.5 | 198 ± 33.2 | 228 ± 69.8 | 0.437 | 0.753 |
| TGL [mg/dL] | 123 ± 51.7 | 131 ± 42.5 | 106 ± 34.2 | 125 ± 48.2 | 0.564 | 0.38 |
| Uric acid [mg/dL] | 5.45 (4.7 - 6.28) | 4.5 (4.08 - 5.62) | 4.7 (4.2 - 5.15) | 4.75 (4.25 - 5.65) | 0.314 | 0.607 |
| MACS [n (%)] | 3 (37.5 %) | 3 (25 %) | 3 (27.3 %) | 1 (7.5 %) | 0.346 | 0.301 |
| Hs-CRP [mg/L] | 3.9 (1.1 – 8.4) | 1.8 (1.1 - 2.2) | 0.8 (0.6 - 1.7) | 1.2 (0.9 – 2.3) | **0.048** | 0.071 |
| 24h SBP [mmHg] | 118 ±5.6 | 125 ± 7.5 | 116 ±9 | 118 ±10.3 | 0.11 | 0.07 |
| 24h DBP [mmHg] | 67.3 ±9.8 | 75.7 ±6.2 | 66.6 ± 5.6 # | 70.7 ±7.1 | **0.026** | 0.155 |
| 24h PR [bpm] | 71.5 ± 8.1 | 73.8 ± 7.9 | 70.9 ± 5.4 | 72.5 ±8.5 | 0.829 | 0.634 |
| Non-dipper status [n (%)] † | 1 (12.5 %) | 1 (14.3%) | 1 (25 %) | 2 (50 %) | 0.374 | 0.131 |
| LVMI [g/m^2^] | 80.9 ±17.4 | 83.9 ±17=9.1 | 77.4 ±16.4 | 88.2 (±24.7) | 0.606 | 0.9 |
| LVH [n (%)] | 1 (12.5 %) | 3 (25 %) | 1 (9.1 %) | 7 (50 %) | 0.115 | 0.502 |
| LAVI [ml/m^2^] | 28.6 ± 10.7 | 23.1 ± 9.2 | 24.7 ± 8.3 | 19.9 ± 7.2 | 0.167 | 0.238 |
| Maximum CIMT [mm] | 1 (0.9 - 1) | 1 (0.8 - 1) | 1 (0.8 - 1.1) | 1 (0.9 -1.1) | 0.787 | 0.372 |
| ASP [n(%)] | 4 (50 %) | 3 (25 %) | 2 (18.2 %) | 4 (28.6 %) | 0.515 | 0.518 |
| SCORE2/-OP [%] ‡ | 9.5 (7.8 – 11.5) | 9.5 (7 - 12.2) | 11 (6 - 15) | 8 (7.25 – 9.75) | 0.572 | 0.12 |
| FHS-ASCVD Risk [%] | 9.7 (8.1- 15) | 11 (5.4 - 13.1) | 7.1 (4.3 – 12.6) | 5.5 (3.7 – 9.3) | 0.118 | **0.042** |

Legend: AI patients were categorized based on catestatin concentrations into those in the lower and upper half, and further into four subgroups (two lowest were identical with first and second quartiles). Data are presented as number (percentage), mean ±standard deviation or median(interquartile range) depending on distribution; p-values were calculated with one-way ANOVA and Tukey's HSD tests (quantitative variables) or Fisher's exact test (categorical variables) with Benjamini-Hochberg correction for multiple testing. Bold font indicates significant p values (<0.05); ***** denotes datapoints significantly different versus those for the ‘very low’ Cts subgroup in post hoc test; # denotes datapoints significantly different versus those for the ‘low Cts’ subgroup in post hoc test; † dipper status was considered only in patients with HT. ‡ Only nondiabetic patients were included in SCORE2/–OP risk estimation (n=11, 11, 16, and 14, for consecutive subgroups). ABPM – ambulatory blood pressure monitoring; ASP – atherosclerotic plaques; BMI – body mass index; bpm – beats per minute; CCA – common carotid artery; CIMT – carotid intima media thickness; DBP – diastolic blood pressure; DMt2 – diabetes mellitus type 2; FHS-ASCVD Risk – 10–year atherosclerotic cardiovascular disease risk calculated based on data from the Framingham Heart Study; HDL-C – high density lipoprotein cholesterol; HT – hypertension; hs–CRP – high sensitivity C–reactive protein; LDL-C – low density lipoprotein cholesterol; LVH – left–ventricular hypertrophy; LVMI – left ventricular mass index; max – maximum; MACS – mild autonomous cortisol secretion; MetS – metabolic syndrome; PPI – proton pump inhibitor; PR – pulse rate; SCORE2/–OP – Systematic Coronary Risk Estimation 2 /–Older People; SBP – systolic blood pressure; TC – Total cholesterol; TGL – triglycerides.
