# Supplementary figures and images for: Low catestatin as a risk factor for cardiovascular disease – assessment in patients with adrenal incidentalomas
Source: Front Endocrinol (Lausanne). 2023 Jul 14;14:1198911. doi: 10.3389/fendo.2023.1198911 (PMC10379641; doi:10.3389/fendo.2023.1198911)

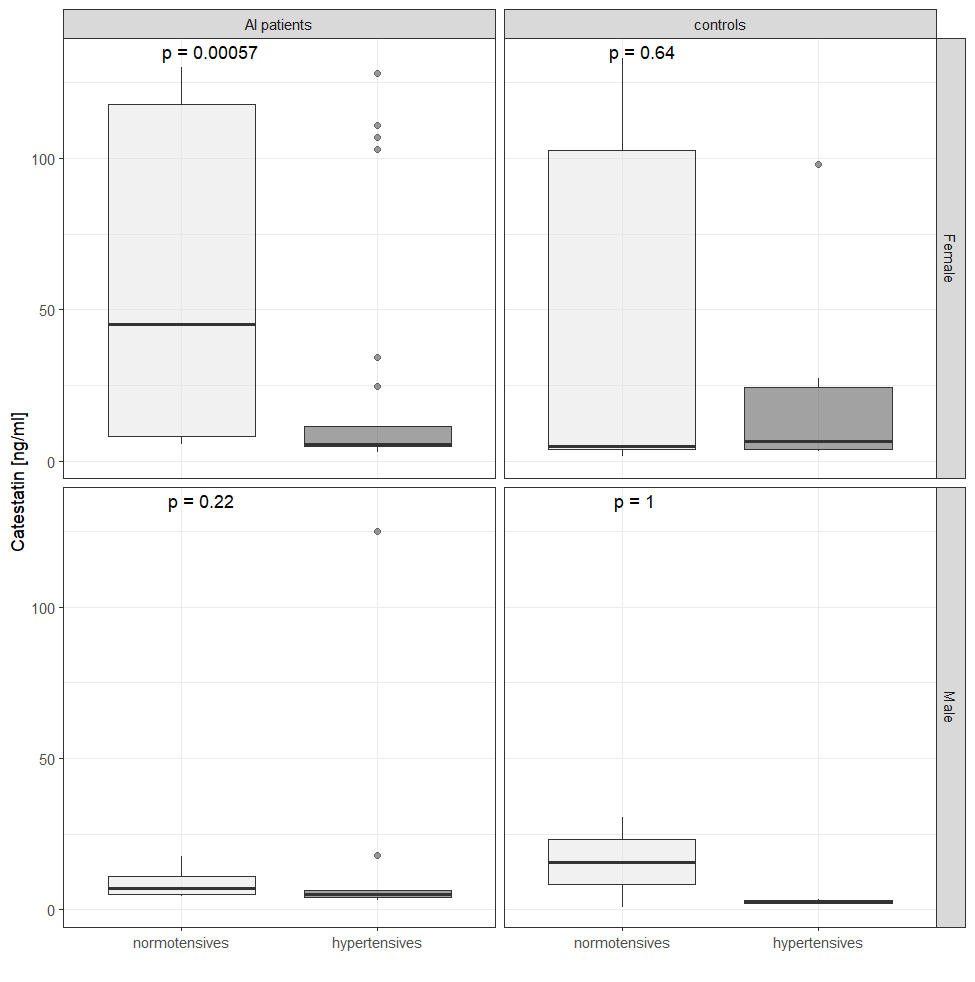

Supplement: Supplementary Figure 1 — Catestatin in normotensive and hypertensive AI patients and controls - analyses in subjects of each sex. Boxplot chart. P-values determined using the Mann-Whitney U test. AI – adrenal incidentaloma. [file Image_1.png]

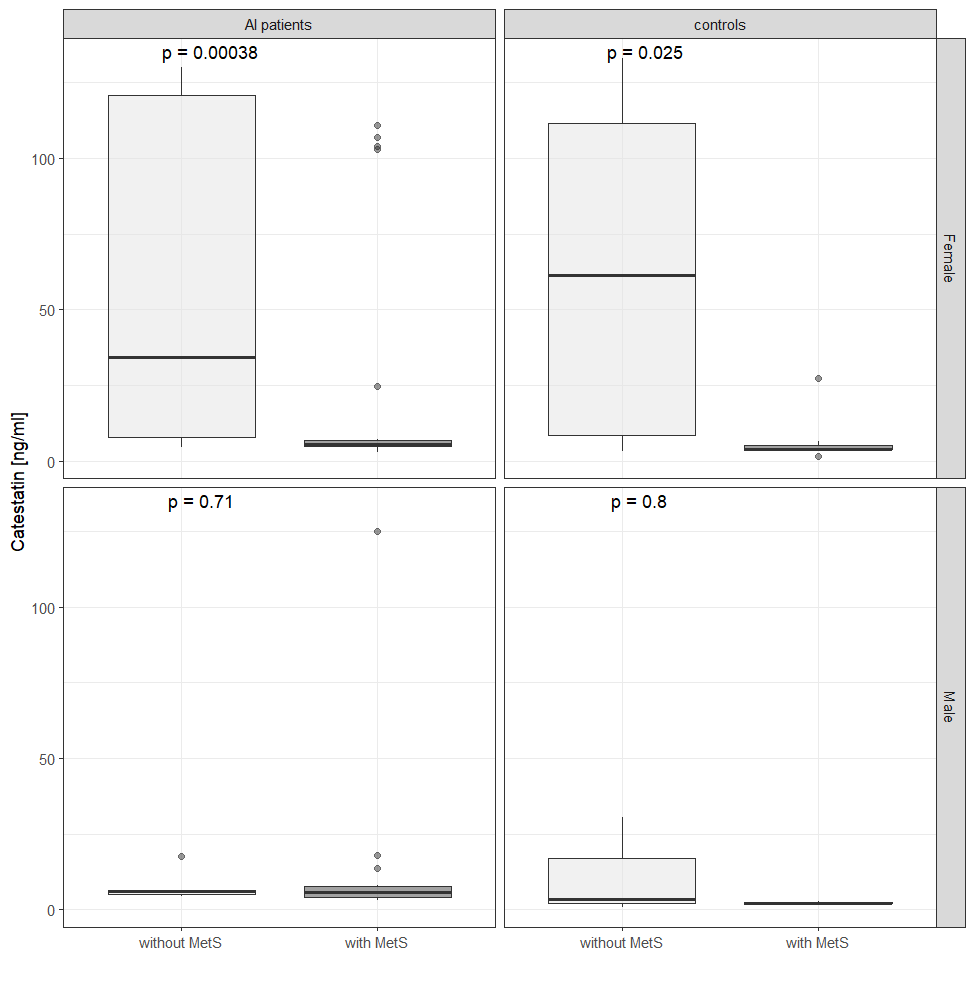

Supplement: Supplementary Figure 2 — Catestatin in subjects without and with metabolic syndrome in controls and AI patients - analyses in subjects of each sex. Boxplot chart. P-values determined using the Mann-Whitney U test. AI – adrenal incidentaloma; MetS – metabolic syndrome. [file Image_2.png]

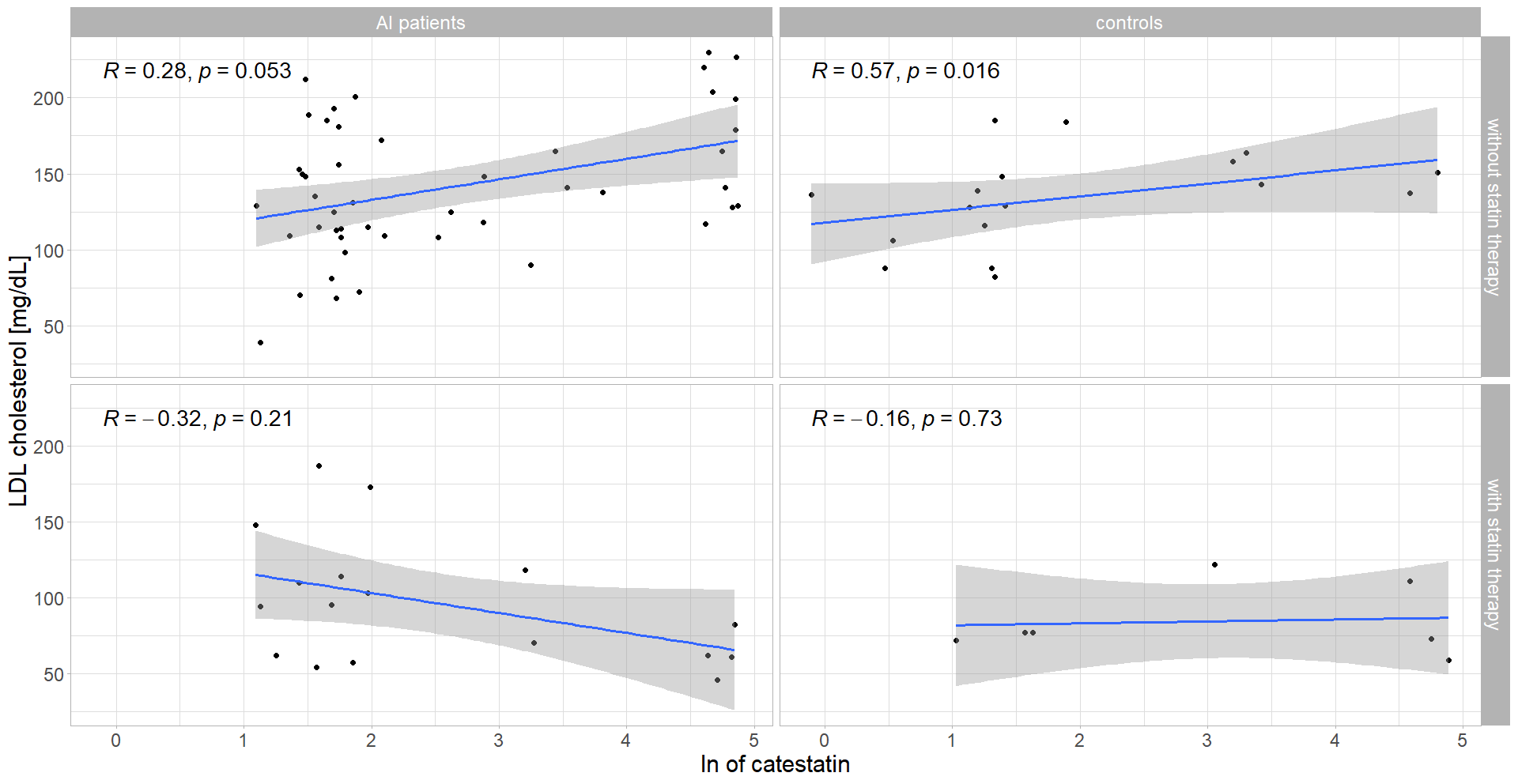

Supplement: Supplementary Figure 3 — Correlations between catestatin and LDL-C level in AI patients and controls. Correlations were computed by the Spearman rank-order method and were tested separately in subjects with and without statin therapy. AI – adrenal incidentaloma, LDL-C – low density lipoprotein cholesterol, ln – natural logarithm. [file Image_3.jpg]

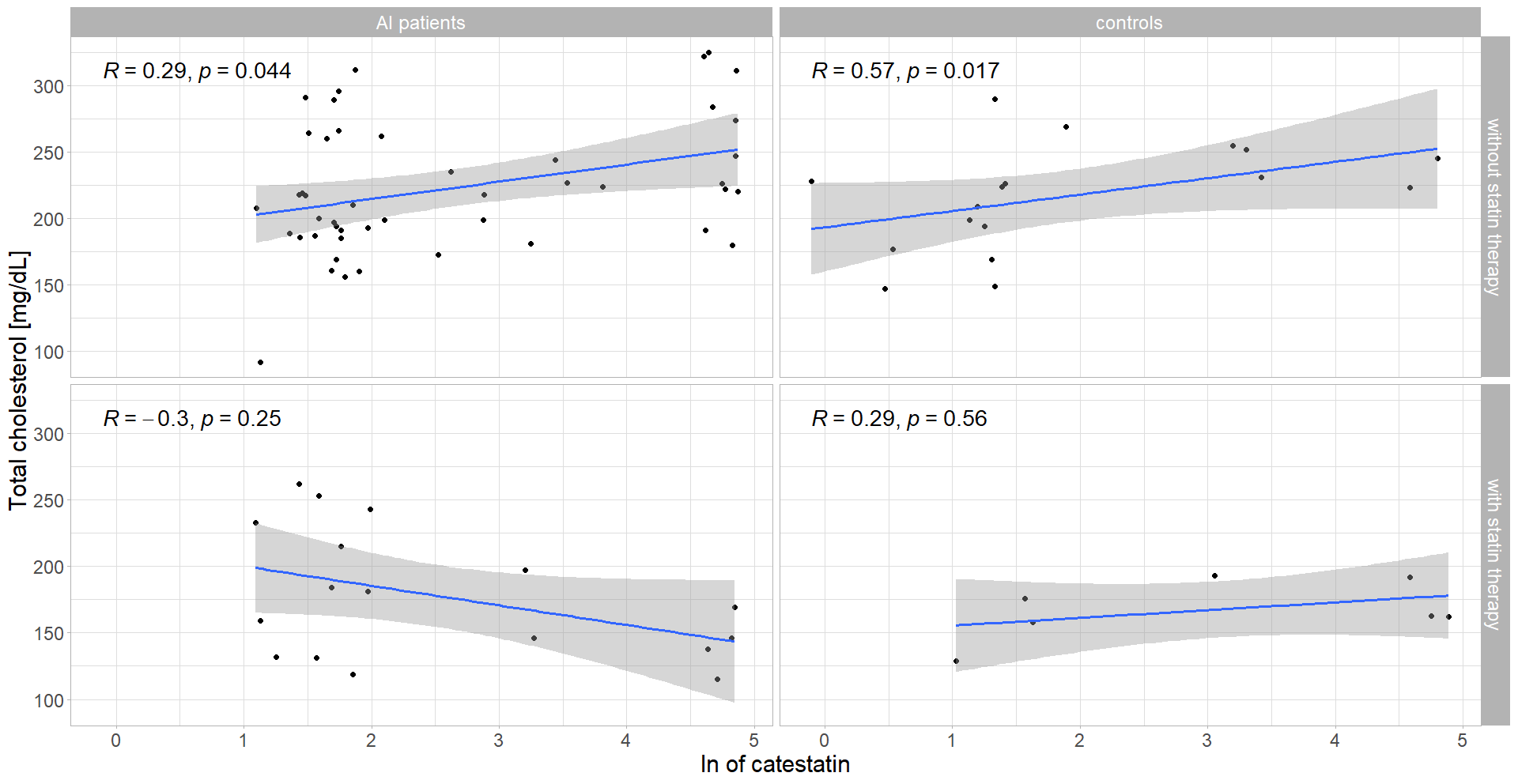

Supplement: Supplementary Figure 4 — Correlations between catestatin and total cholesterol level in AI patients and controls. Correlations were computed by the Spearman rank-order method and were tested separately in subjects with and without statin therapy. AI – adrenal incidentaloma, ln – natural logarithm. [file Image_4.jpg]
